# Composition of breast milk from mothers of premature and full-term infants and its influence in Z-Scores for infant physical growth

**DOI:** 10.1186/s12887-024-04757-4

**Published:** 2024-04-30

**Authors:** Guixia Chen, Rongxian Xu, Jiyong Zhang, Meifeng Yang, Jianxia Fan, Yinying Huang, Xiaoling Sun

**Affiliations:** 1 Department of Child Healthcare, Women and Children’s Hospital, School of Medicine, Xiamen University/Xiamen Maternal and Child Healthcare Hospital, Xiamen, China; 2 Department of Nutrition and Food Hygiene, School of Public Health, Fujian Medical University, Fuzhou, China; 3 Nursing Department, Women and Children’s Hospital, School of Medicine, Xiamen University/Xiamen Maternal and Child Healthcare Hospital, Xiamen, China

**Keywords:** Breast milk analysis, Milk, Nitrogen compounds, Infant, Growth, Non-protein nitrogen

## Abstract

**Background:**

Breast milk contains various crucial nutrients and biologically active substances and is ideal for newborns. This study aimed to analyze the composition of breast milk from mothers of premature and full-term infants and its influences on the growth of infants.

**Methods:**

Infant-mother dyads examined at our Hospital (March 2016 to May 2017) were included. Milk was collected at 0–1 month, 2–3 months, and 5–6 months and analyzed using a MIRIS human milk analyzer. Z-scores of weight-for-length (WLZ), weight-for-age (WAZ), and length-for-age (LAZ) were calculated.

**Results:**

This study included full-term (> 37 weeks of gestation, *n* = 177) and premature (< 37 weeks, *n* = 94) infant-mother dyads. The premature infants showed higher ΔWAZ, ΔLAZ, and ΔWLZ from infancy to toddlerhood for the physical growth speed, compared with term infants (*P* < 0.001). All proteins and true protein components of breast milk decreased with infants’ age (*P* < 0.001). For premature and full-term infants, differences in ΔWAZ and ΔLAZ from birth to infancy and the difference in ΔLAZ, WAZ, and LAZ in toddlerhood were positively associated with non-protein nitrogen (NPN) (all *P* < 0.05), while the Z-score differences in ΔWLZ from birth to infancy were negatively associated with NPN (all *P* < 0.05). For premature babies, from birth to infancy stage, ΔWAZ was positively correlated with NPN and carbohydrates while negatively correlated with dry matter (all *P* < 0.05), and ΔLAZ correlated with NPN (β = 0.428, *P* = 0.005).

**Conclusion:**

Breastfeeding helped premature infants compensatory growth when compared to term infants. Whileduring early infancy stage ΔWLZ gain was negatively associated with increased amounts of NPN in breast milk. This might mean although NPN increase the Z-scores of weight-for-age and length-for-age, with no rise in adipose tissue mass.

**Supplementary Information:**

The online version contains supplementary material available at 10.1186/s12887-024-04757-4.

## Background

Breast milk is the ideal food for newborns and is recommended by the World Health Organization (WHO) [1]. Direct breastfeeding is naturally beneficial to infants [2, 3]. Breast milk is an evolution-shaped mixture of essential nutrients, energy sources, and bioactive compounds that provide energy, allow development, and protect against infectious and non-communicable diseases [4, 5].

The composition of breast milk is not easily affected by race [6], while maternal diet is a major determinant of the quality of breast milk [7]. Nevertheless, due to different diets and physical conditions, the content, composition, and active constituents of fat in breast milk can be slightly different [6, 8].

Differences in the composition of breast milk directly affect the growth and development of infants [9–11]. Systematic reviews by Eriksen et al. [12] and Reyes et al. [11] highlighted that high-quality studies on the relationship between the composition of breast milk and the growth of infants were sparse. A review by Lind et al. [13] concluded that the results of the available studies were inconsistent. Prentice et al. [14] showed that the percentages of fat and carbohydrate in breast milk were associated with gains in weight, body mass index (BMI), and adiposity between three and 12 months of age. The ongoing Cambridge Baby Growth and Breastfeeding Study will help answer those conflicting results [15]. Visuthranukul et al. reported that, by the age of 2, small-for-gestational-age (SGA) premature infants who exclusively received human milk exhibited more significant compensatory growth, with no concurrent increase in adipose tissue mass, in comparison to appropriate-for-gestational-age (AGA) infants [16].

This study aimed to explore the relationship between the composition of breast milk and an infant’s physical growth.

## Methods

### Study design and patients

This retrospective study included full-term and premature infants and their mothers from the Child Healthcare Department of Xiamen Maternity and Child Health Hospital between March 2016 and May 2017.

Full-term infants referred to those with a gestational age of ≥ 37 weeks; otherwise, they were considered premature infants. Infants with severe complications (including serious heart and kidney diseases, such as myocarditis, various cardiomyopathies, and chronic kidney disease), serious infections (such as septicemia), or known severe congenital malformations (such as digestive tract malformations, cyanotic congenital heart disease, etc.) were excluded.

The study was approved by the Ethics Committee of Xiamen Maternity and Child Health Hospital (approval number KY-2021-038-K02). Informed consent was waived because of the retrospective nature of the study.

### Breast milk analysis

**Fig. 1:**
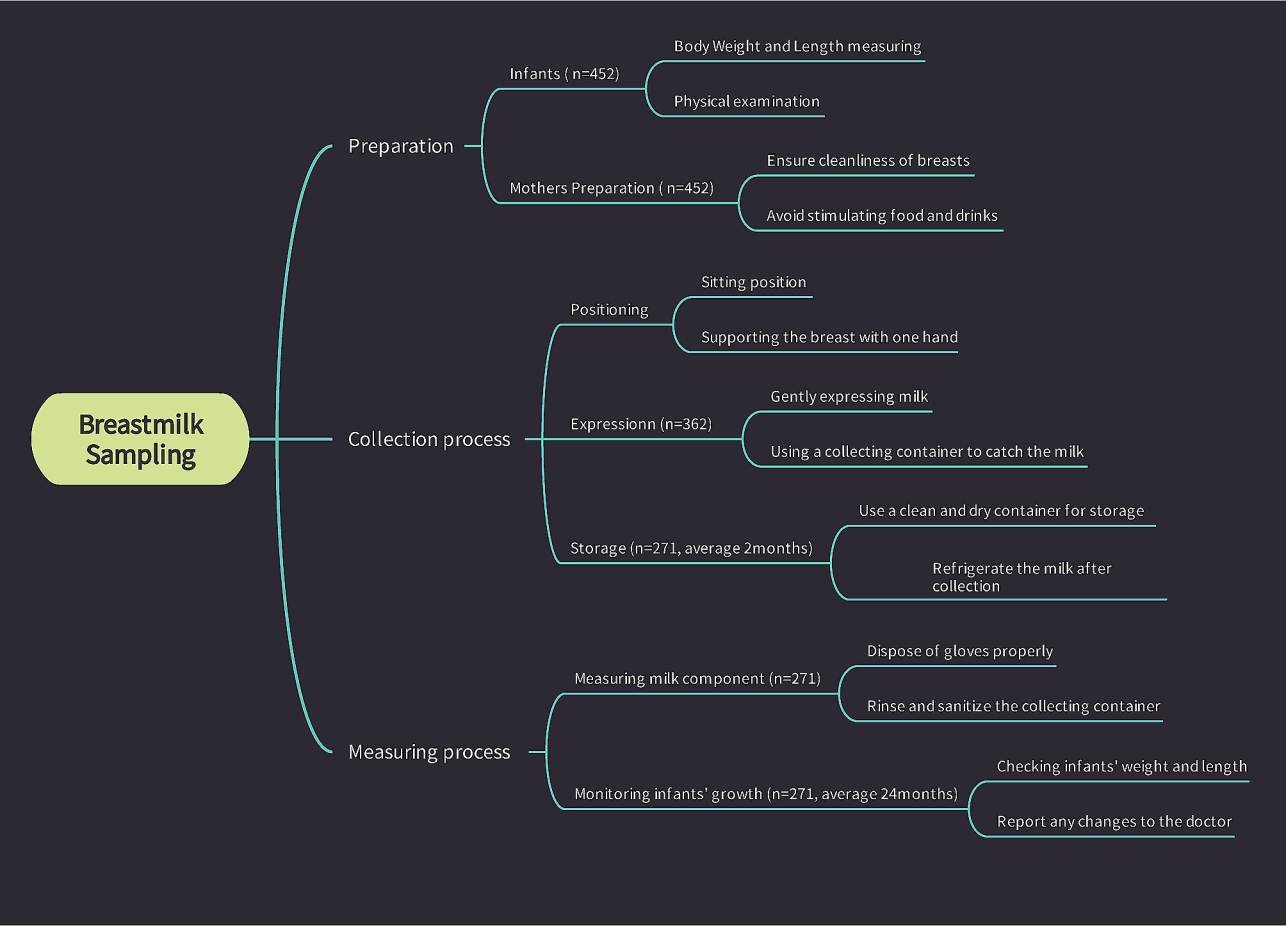
Child health clinic breast milk analysis convenient sampling process flowchart

All sample collection was conducted during outpatient physical examinations at 0–1 month, 2–3 months, and 4–6 months (actual age) (Fig. 1). As per routine procedure at the authors’ hospital and during well baby check, before breastfeeding, the mother collected 5–10 ml of breast milk from one side of the breast by hand milking into a sterile tube and then stored it in a refrigerator at 4 °C for testing on the same day. A MIRIS HMA (Human Milk Analyzer, Miris, Uppsala, Sweden) was used to determine the contents of protein, fat, carbohydrate, dry matter (defined as solid substances in the milk excluding liquid parts), and energy. The protein content is mainly determined by nitrogenous substances. The nitrogen content in breast milk includes nitrogen protein (NP) and non-protein nitrogen (NPN). The true protein (TP) refers to the protein nitrogen except for NPN. In this study, the total protein and TP were detected, and the difference between the two was NPN.

### Physical examination

In the children’s healthcare department, qualified physicians and nurses performed physical examinations on children as part of routine postpartum care. The body indexes were determined using Z-scores after length and weight were measured [17], including the weight-for-age Z-score (WAZ), length-for-age Z-score (LAZ), weight-for-length Z-score (WLZ), the difference among different stage weight-for-age Z-scores (ΔWAZ), the difference among different stage length-for-age Z-scores (ΔLAZ), and difference among different stage weight-for-length Z-scores (ΔWLZ). According to the 2006 WHO Child Growth Standards [18], Z-scores for body length = (measured body length - reference median (M))/standard deviation (SD); Z-score for weight = (measured body weight - M)/SD; Z-scores for weight for length = (measured weight for length - M)/SD. The differences among Z-score for length and weight of the newborns at birth, infancy stage (average of 2.0 months), and toddlerhood stage (average of 24.5 months), and the growth indicators of ΔWAZ, ΔLAZ, and ΔWLZ were analyzed. For the preterm infants, actual age was used. “Exclusive breastfeeding” was defined as 100% breastfeeding, until to nearly 6 months.

### Sample size

Considering the 5–10 events per variable (EPV) guideline, with 12 independent variables in our study, the minimum sample size required ranges from 60 to 120 [19].

### Statistical analysis

SPSS 22.0 (IBM, Armonk, NY, USA) was used for data analysis. Continuous variables that conform to a normal distribution were expressed as mean ± standard deviation, while those that do not conform to a normal distribution were expressed as median (P25, P75). The t-test/Mann-Whitney U test, analysis of variance (ANOVA) /Kruskall-Wallis H test, and linear regression analysis were used to compare the groups. Differences with two-sided P-values < 0.05 were considered statistically significant.

## Results

### Characteristics of the patients

**Fig. 2:**
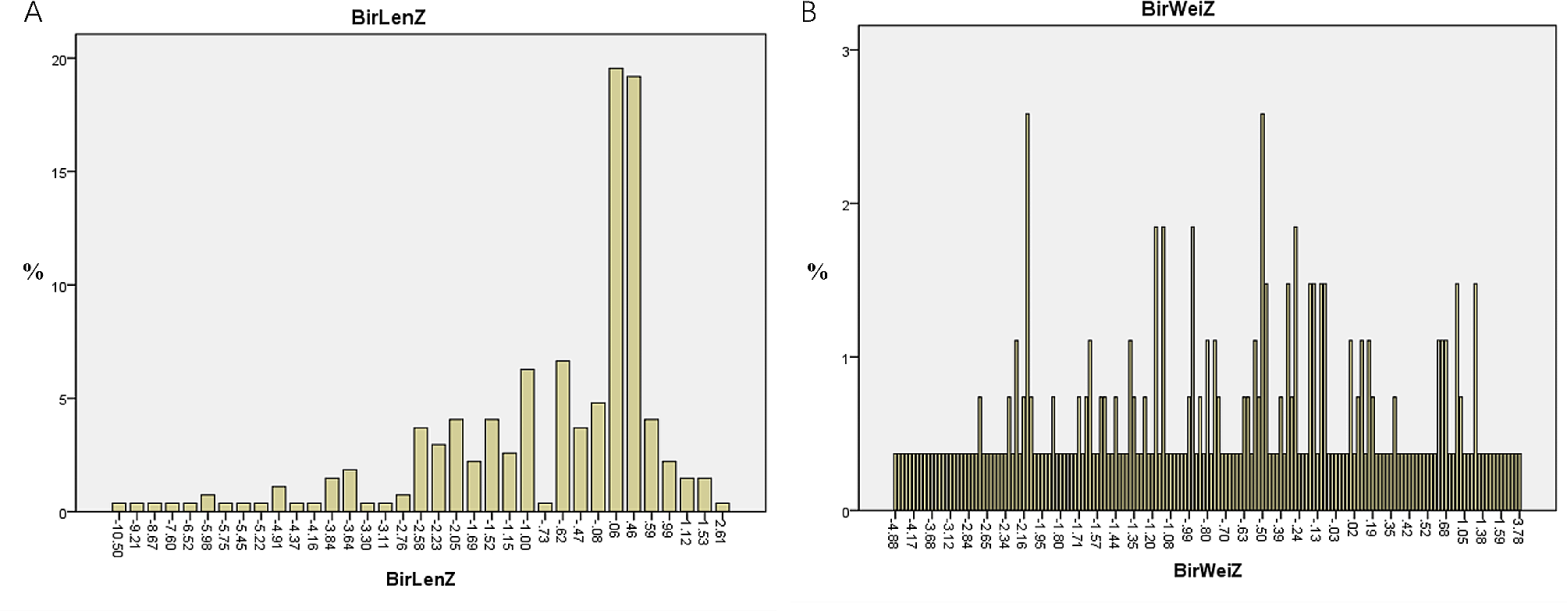
The z-scores at time of birth (**A** length; **B** weight)

This study included 177 full-term infants with an average gestational age of 39.3 ± 1.1 weeks, a weight of 3.21 ± 0.45 (range, 1.95-5.00) kg, body length of 49.5 ± 1.6 cm, and a male-to-female ratio at 88:89. This study also included 94 premature infants with an average gestational age of 34.7 ± 2.5 weeks, a weight of 2.23 ± 0.54 (range, 0.95–3.20) kg, body length of 45.1 ± 4.0 cm, and a male-to-female ratio of 50:44. Their mothers were 20–44 years old, enjoyed a stable life, and practiced light physical activities. They all received dietary guidance during pregnancy and lactation and provided breast milk at a median of 0 (range, 0–3) days after birth (94.3% for full-term infants and 79.3% for premature infants). Some mothers had to delay nursing for up to 31 days for their reasons and health problems, but the babies were healthy and had no special diseases. The proportion of full-term infants who exclusively received breast milk within the past week was 93.3%, while the proportion of premature infants was 74.5%. The remaining infants were mainly but not exclusively fed with breast milk (Table 1). Figure 2 displays the Z-scores.

**Table 1:** Characteristics of the patients

Characteristics	Full-term (*n* = 177)	Premature (*n* = 94)	*P*
Gestational age (weeks), mean ± SD	39.3 ± 1.1	34.7 ± 2.5	< 0.001
Sex (M/F)	88/89	50/44	0.588
Birth weight (kg), mean ± SD	3.21 ± 0.45	2.23 ± 0.54	< 0.001
Birth length (cm), mean ± SD	49.5 ± 1.6	45.1 ± 4.0	< 0.001
Mother’s age (years), mean ± SD	29.8 ± 4.1	30.4 ± 3.8	0.255
Start time for breastfeeding (day)	0 (0–3)	0 (0–3)	> 0.999
Rate of exclusive breastfeeding (male/female)	83/82 (93.2%)	37/33 (74.5%)	< 0.001

### Composition analysis of the breast milk

Table 2 shows that the fat, carbohydrate, dry matter, and energy were not different among the three infant age groups (all *P* > 0.05), while all proteins and true proteins decreased with infant age (both *P* < 0.001).

**Table 2:** Comparisons of the nutrients in breast milk from both term and preterm mothers of infants at different age

Composition, g/100mL	0–1 months (*n* = 185)	2–3 months (*n* = 56)	4–6 months (*n* = 30)	*P*
Fat	4.0 (3.0, 5.1)	4.0 (2.6, 5.2)	4.2 (3.2, 5.3)	0.886
AlProt	1.2 (1.1, 1.4)	1.0 (0.9, 1.1) ^*^	0.8 (0.8, 1.0) ^*#^	< 0.001
TruPro	1.0 (0.9, 1.1)	0.8 (0.7, 0.9) ^*^	0.7 (0.7, 0.8) ^*#^	< 0.001
Carbohydrate	7.9 (7.6, 8.2)	7.8 (7.6, 8.1)	8.0 (7.7, 8.1)	0.268
Dry matter	13.3 (12.4, 14.4)	12.9 (11.7, 14.1)	13.1 (12.1, 14.0)	0.414
AllEner	74.0 (65.0, 84.0)	72.0 (58.2, 83.5)	74.5 (64.8, 84.2)	0.851

^*^P < 0.05 vs.0–1 Month of Age

^#^*P* < 0.05 vs. 2–3 Months of Age

### Differences in growth between premature and full-term infants

Table 3 shows the independent-sample Mann-Whitney nonparametric test and compared with full-term infants. Premature infants had higher ΔLAZ _infancy stage-birth_ (1.22 vs. 0.22, *P* < 0.001), ΔWAZ _infancy stage-birth_ (1.63 vs. 0.52, *P* < 0.001), ΔWLZ _infancy stage-birth_ (0.07 vs.0.02, *P* < 0.001), ΔLAZ _toddlerhood-infancy stage_ (0.70 vs. -0.85, *P* < 0.001), ΔWAZ _toddlerhood-infancy stage_ (1.01 vs. -0.32, *P* < 0.001), and ΔWLZ _toddlerhood-infancy stage_ (0.04 vs. 0.00, *P* < 0.001).

**Table 3:** Changes of ΔZ for the physical growth of premature and full-term infants from infancy stage to toddlerhood stage

	Full Term (*n* = 177)	Premature (*n* = 94)	*P*
Infant to birth ΔLAZ	0.218 (-0.340-0.986)	1.219 (0.377–2.132)	< 0.001
Infant to birth ΔWAZ	0.517 (-0.179-1.151)	1.629 (0.828–2.195)	< 0.001
Infant to birth ΔWLZ	0.018 (-0.027-0.083)	0.065 (0.000-0.144)	< 0.001
Toddler to infant ΔLAZ	-0.854 (-1.828-0.042)	0.702 (-0.830-1.898)	< 0.001
Toddler to infant ΔWAZ	-0.323 (-1.248-0.300)	1.011 (0.301–1.927)	< 0.001
Toddler to infant ΔWLZ	0.000 (-0.051-0.044)	0.039 (0.000-0.114)	< 0.001

ΔWAZ: difference among weight-for-age Z-scores; ΔLAZ: difference among length-for-age Z-scores; ΔWLZ: difference among weight-for-length Z-scores

Composition analysis of the breast milk between full-term and premature infants.

The total proteins and true proteins of breast milk for both term and premature infants’ decreased with months. While the breast milk composition was compared between full-term and premature infants, there were no significant differences (all *P* > 0.05) (Table 4).

**Table 4:** Composition analysis of breast milk from mothers of premature and full-term infants

Composition, g/100 mL	0–1 months			2–3 months		4–6 months		P
	Group	n	Median (P25, P75)	n	Median (P25, P75)	n	Median (P25, P75)	
Fat	Full-term	118	4.1 (2.98, 5.03)	41	3.9 (2.85, 5.15)	18	3.9 (3.15, 5.43)	0.843
	Premature	67	3.9 (3.1, 5.2)	15	4.2 (2.1, 5.2)	12	4.2 (3.2, 4.6)	0.998
Total proteins	Full-term	118	1.2 (1.1, 1.4)	41	1.0 (0.9, 1.1)	18	0.8 (0.8, 1)	< 0.001
	Premature	67	1.2 (1.1, 1.4)	15	1.0 (0.9, 1.2)	12	0.8 (0.7, 1.0)	< 0.001
True proteins	Full-term	118	1.0 (0.9, 1.1)	41	0.8 (0.7, 0.9)	18	0.7 (0.7, 0.8)	< 0.001
	Premature	67	1.0 (0.9, 1.2)	15	0.8 (0.7, 1.0)	12	0.7 (0.6, 0.9)	< 0.001
Carbohydrate	Full-term	118	7.9 (7.6, 8.2)	41	7.8 (7.5, 8.1)	18	8.0 (7.8, 8.1)	0.058
	Premature	67	7.9 (7.6, 8.1)	15	8.1 (7.7, 8.2)	12	7.9 (7.6, 8.0)	0.859
Dry matter	Full-term	118	13.4 (12.48, 14.4)	41	12.7 (11.7, 14.0)	18	13.2 (12.1, 14.3)	0.395
	Premature	67	13.2 (12.4, 14.7)	15	13.0 (11.7, 14.2)	12	13.1 (12.2, 13.7)	0.808
Energy	Full-term	118	74.5 (65.0, 83.3)	41	72.0 (60.0, 83.0)	18	73.0 (64.5, 85.2)	0.813
	Premature	67	73.0 (66.0, 85.0)	15	75.0 (58.0, 84.0)	12	74.5 (65.3, 79.3)	0.965

### Breast milk composition and infant physical growth

The results of the physical examinations in the infancy stage (average of 2.0 months) and toddlerhood stage (average of 24.5 months) were presented. Since 61.6% of the infants were < 24 months, the “height (length)” was uniformly expressed as “length” for short. The results showed that the changes of Z-scores for the physical growth from infancy stage to toddlerhood stage were associated with non-protein nitrogen (NPN) in the breast milk (WAZ _infancy_: β = 0.205, *P* = 0.019; LAZ _toddlerhood_: β = 0.227, *P* = 0.009; ΔWAZ _toddlerhood-infancy stage_: β = 0.255, *P* = 0.004; ΔLAZ _toddlerhood-infancy stage_: β = 0.286, *P* = 0.001; ΔWAZ _infancy stage-birth_: β = 0.220, *P* = 0.013; ΔWLZ _infancy stage-birth_: β=-0.178, *P* = 0.045) (Table 5).

**Table 5:** Relation between NPN in the breast milk and Z-scores for the physical growth of both premature and full-term infants

	Non-standardized coefficient B	Standard error	Standardized coefficient beta	*P*
Infancy stage WAZ	5.718	2.428	0.205	0.019
Toddler stage LAZ	20.479	7.824	0.227	0.009
Toddler to infant ΔWAZ	7.915	2.721	0.255	0.004
Toddler to infant ΔLAZ	25.389	7.665	0.286	0.001
Infant to birth ΔLAZ	4.955	1.990	0.220	0.013
Infant to birth ΔWLZ	-0.373	0.185	-0.178	0.045

WAZ: weight-for-age Z-score; LAZ: length-for-age Z-score; WLZ: weight-for-length Z-score; ΔWAZ: difference among weight-for-age Z-scores; ΔLAZ: difference among length-for-age Z-scores; ΔWLZ: difference among weight-for-length Z-scores

In full-term infants, the changes of Z-scores for the physical growth of the infants from infancy stage to toddlerhood stage were associated with NPN in the breast milk (WAZ _infancy_: β = 0.390, *P* < 0.001; LAZ _infancy_: β = 0.397, *P* < 0.001; ΔWAZ _toddlerhood-infancy stage_: β = 0.284, *P* = 0.011; ΔLAZ _toddlerhood-infancy stage_: β = 0.379, *P* = 0.001; ΔWLZ _infancy stage-birth_: β=-0.264, *P* = 0.018) (Table 6).

**Table 6:** Relation between NPN in the breast milk and Z-scores for the physical growth of full-term infants after breastfeeding

	Non-standardized coefficient B	Standard error	Standardized coefficient beta	*P*
Toddler stage WAZ	10.635	2.918	0.39	< 0.001
Toddler stage LAZ	34.779	9.362	0.397	< 0.001
Toddler to infant ΔWAZ	7.877	3.057	0.284	0.011
Toddler to infant ΔLAZ	32.744	9.291	0.379	0.001
Infant to birth ΔWLZ	-0.567	0.238	-0.264	0.018

WAZ: weight-for-age Z-score; LAZ: length-for-age Z-score; ΔWAZ: difference among weight-for-age Z-scores; ΔLAZ: difference among length-for-age Z-scores; ΔWLZ: difference among weight-for-length Z-scores

In premature infants, ΔWAZ _infancy stage-birth_ and ΔLAZ _infancy stage-birth_ correlated with NPN (β = 0.362, *P* = 0.017), carbohydrate (β = 1.921, *P* = 0.031), and dry matter (β=-10.147, *P* = 0.016), and ΔLAZ _infancy stage-birth_ correlated with NPN (β = 0.428, *P* = 0.005) (Table 7).

**Table 7:** Relation between compositions of breast milk and changes of Z-scores for the physical growth of premature infants

		Non-standardized coefficient B	Standard error	Standardized coefficient beta	*P*
ΔWAZ*	NPN	6.423	2.643	0.362	0.017
	Carbohydrate	4.609	2.102	1.921	0.031
	Dry matter	-6.254	2.557	-10.147	0.016
ΔLAZ*	NPN	12.332	4.263	0.428	0.005

* Refers to the difference of Z-scores for physical growth between the infancy stage and at birth

## Discussion

Breast milk is rich in nutrients, which might affect the growth of babies. The mothers of infants with different gestational ages or months of age produce different compositions of breast milk. Therefore, this study aimed to analyze the composition of breast milk from mothers of premature and full-term infants and its influences on the growth of infants. NPN positively impacts the physical growth of infants.

In this study, the total protein, true protein, and NPN of breast milk from the mothers of premature or full-term infants for the age of 0–6 months presented a declining trend, and significant differences were observed between groups. Mothers produced breast milk with suitable protein composition to satisfy the nutritional demand of the infants [2, 5]. There were no differences in breast milk nutrients from the mothers of premature infants compared with full-term infants. The present study showed that the ΔWAZ, ΔLAZ, and ΔWLZ showed that premature infants grew faster than full-term infants. Still, the rate of exclusive breastfeeding was lower in the premature group (74.5% vs. 93.2%), and the increased utilization of preparations, including human milk fortifier, might, at least partially, account for the observed enhanced weight and length gain, aiding in catching up within the premature group [20].

Dietary protein intake impacts the protein content in breast milk [21–23]. Binder et al. [7] reported that maternal diet is a major determinant of the quality of breast milk. Protein intake by the newborn is directly related to neurodevelopment [24], especially lactoferrin [25]. Preterm infants require twice the amount of proteins than term infants [26, 27], while a too-high protein content in the first months of life in full-term infants is a risk factor for childhood obesity [28]. Therefore, the mothers must follow a proper diet to ensure their milk contains adequate proteins [29]. Protein is one of the key nutrients for the growth and brain development of premature infants. In this study, the average protein content in breast milk from the mothers of premature infants at the age of 0 months was similar to the results in the first week of the study by Kreissl et al. [30]. The protein content in breast milk from the mothers of full-term infants was similar to Gao et al. [31].

The protein content is mainly determined by nitrogenous substances. The nitrogen content in breast milk includes NP and NPN, of which the former accounts for 95% and the latter about 5%. The main nitrogenous substance in breast milk is protein, and other nitrogenous substances are mainly derived from ammonia, carbamide, creatine, creatinine, uric acid, orotic acid, peptides, hippuric acid, and amino acids, which are called NPN. The TP refers to the protein nitrogen except for NPN. In this study, the total protein and TP were detected [32], and the difference was NPN. This study showed that NPN was closely related to infant growth. Those amino acids in NPN might be free amino acids, such as taurine, which is the most abundant constituent in the milk of many species. The free amino acid pool of human milk and other species has generally been disregarded because the proportion of these amino acids compared to the amino acids in the milk protein is quite small [33].

The NPN in breast milk had significant positive impacts on the changes of Z-scores for length and weight of physical examination in infancy and toddlerhood stages. The calculation of the ΔZ can be helpful for the longitudinal comparison of the growth status of babies with a larger span of months of age. The NPN in breast milk was positively associated with the WAZ and WLZ. The amino acids, peptides, orotic acid, and creatine in breast milk may be helpful for infant growth and development. The NPN was negatively correlated with the difference of Z-scores for weight for length. According to a meta-analysis, direct breastfeeding reduces the risk of obesity in children between 0 and 6 years old in China [34], compared with bottle feeding, as observed elsewhere in the world [13, 14, 35].

This study showed that the nutrients in breast milk were not different between premature and full-term birth, but the rate of exclusive breastfeeding was lower in premature neonates compared with term ones. It is generally difficult for neonatal intensive care unit (NICU) infants to allow direct breastfeeding, but the breastfeeding rate was increased through quality improvement measures for breastfeeding in the NICU [36]. In order to achieve the 2025 global breastfeeding target [37], it is necessary to understand that breastfeeding is equally important for premature infants. Based on 24-h dietary recall, only 24.4% of the mothers practiced exclusive breastfeeding in the United States of America [37]. Therefore, much remains to be done to promote breastfeeding globally [37].

This study has limitations. It was a retrospective study that used clinical data. The data that could be analyzed were limited to those available in the charts. The data available in the charts were from foremilk. It is well-known that the composition of breast milk changes during the day and between foremilk and hindmilk [5, 38]. There is also a risk of misclassification bias. There is a risk of confounding factors that were not indicated in the charts and could not be controlled for. The breast milk was analyzed whole in a routine clinical setting, and it was not defatted, which can influence the protein content [39]. Still, all samples from both groups were treated in the same way, and the groups should still be comparable. The breast milk was not analyzed exactly at the same time points with respect to birth, which could introduce some bias. In addition, breast milk composition can change during the day, and a single sample might not be enough to determine the real composition [40]. Since many women were directly feeding their babies, it was impossible to document the amount of milk ingested by the babies. We also acknowledge that we did not perform sample size calculations prior to initiating the study, which raises concerns about the statistical robustness of the current sample size.

## Conclusions

In conclusion, all proteins and true protein components of breast milk were decreased with months. Although the content of NPN was a small part of breast milk, it played an important role in decreasing the growth of ΔWLZ in the early infancy stage as well as improving ΔWAZ and ΔLAZ until the toddlerhood stage. These results might serve as a reference to determine or evaluate the small molecules in NPN of breast milk that help improve growth and prohibit early overweight for infants.

### Electronic supplementary material

Below is the link to the electronic supplementary material.

Supplementary Material 1

## Data Availability

All data generated or analyzed during this study are included in this published article.

## Abbreviations

WHO: World Health Organization
WAZ: Weight-for-age Z-score
LAZ: Length-for-age Z-score
WLZ: Weight-for-length Z-score
ANOVA: Analysis of variance
TP: True protein
NICU: Neonatal intensive care unit
NPN: Non-protein nitrogen
