# Supplementary material for: Composition of breast milk from mothers of premature and full-term infants and its influence in Z-Scores for infant physical growth
Source: BMC Pediatr. 2024 Apr 30;24:292. doi: 10.1186/s12887-024-04757-4 (PMC11059756; doi:10.1186/s12887-024-04757-4)
Supplement: Supplementary file 1 — Supplementary Material 1 [file 12887_2024_4757_MOESM1_ESM.docx]

**Supplement Table 1** Comparisons of the nutrients in breast milk between full-term and preterm mothers of infants at 1 month.

|  | Full-term (n=118) | Premature (n=67) | P |
| --- | --- | --- | --- |
| Fat | 4.11±1.62 | 4.24±1.79 | 0.605 |
| AlProt | 1.25±0.18 | 1.24±0.24 | 0.733 |
| TruPro | 1.03±0.15 | 1.02±0.21 | 0.733 |
| Carbohydrate | 7.87±0.43 | 7.83±0.43 | 0.896 |
| Dry matter | 13.44±1.46 | 13.51±1.68 | 0.570 |
| AllEner | 75.00±14.21 | 74.05±18.05 | 0.673 |
